# A Validated Method for Cholesterol Determination in Turkey Meat Products Using Relative Response Factors

**DOI:** 10.3390/foods8120684

**Published:** 2019-12-15

**Authors:** Simona Grasso, Sabine M. Harrison, Frank J. Monahan, Nigel P. Brunton

**Affiliations:** 1 School of Agriculture, Policy and Development, University of Reading, Reading RG6 6AR, UK; simona.grasso@ucdconnect.ie; 2 School of Agriculture and Food Science, University College Dublin, Dublin 4, Ireland; sabine.harrison@ucd.ie (S.M.H.); frank.monahan@ucd.ie (F.J.M.)

**Keywords:** cholesterol, relative response factor, turkey meat, matrix effect, recovery, linearity, precision, accuracy

## Abstract

The objective of this study was to develop a precise and accurate method to quantify cholesterol in turkey meat products using relative response factors, based on a modification of a previously published method for plant sterols determination. Validation was performed using neat solutions to determine linearity, precision, and accuracy. The method was linear in the concentration range considered (1–20 µg/mL, *r*^2^ ≥ 0.991). Precision and accuracy were within the acceptability guidelines of the U.S. Food and Drug Administration (FDA) for method validation (<20% relative standard deviation (RSD) at the lower limit of quantification (LLOQ) and <15% RSD for other standards). Turkey meat was spiked with cholesterol at two levels (low = 3 µg/mL and high = 18 µg/mL), either before or after saponification, to establish the recovery and matrix effects. Recovery ranged from 94% to 105%, with a mean value of 105% at the low spike level and 95% at the high spike level. No significant matrix effects were found (90% to 112% recovery). This method is reliable for the quantification of cholesterol in turkey meat products in the range 0.4–8 mg/g.

## 1. Introduction

High blood cholesterol is a long-established contributory risk factor to the development of heart disease [1]. Cholesterol content must always appear on U.S. nutrition labels regardless of amounts present in the food [2]. It is therefore important to develop accurate and reliable methods for rapid cholesterol measurement in foods.

The official gas chromatographic (GC) method used for cholesterol determination in foods is the Association of Official Analytical Chemists (AOAC) 994.10 [3]. Many analysts consider this method to be lengthy and labour intensive as a result of the large amount of chemicals needed, the complexity of the method, and the several preparatory steps needed before GC analysis [4]. For these reasons, many authors have developed their own methods for cholesterol determination in meat and other food matrices since the publication of AOAC 994.10 [4,5,6]. However, the majority of the methods developed for meat and other foods rely on external calibration rather than internal standards for quantification purposes. For example, no publications to date have used relative response factors (RRFs) to validate GC cholesterol methods, although the use of RRFs has some advantages over traditional methods relying on calibration curves. The response factors of compounds in GC can vary from one day to another and also from one instrument to another [7]. To be able to consider these variations, an external calibration curve is usually produced for each run. However, RRFs are not affected by day to day variations (i.e., are constant) and there is no need to run a calibration curve with every run.

In this context, the aim of the work was to develop a precise and accurate method to quantify cholesterol in turkey meat products using RRFs based on a modification to an existing procedure for plant sterols (Grasso et al. [8]), as well as testing cholesterol recovery and matrix effects using a turkey meat product. The validation was evaluated to fall within the guidelines of the U.S. Food and Drug Administration (FDA) [9].

## 2. Materials and Methods

### 2.1. Chemicals

Cholesterol standard (purity 98%), the internal standard (ISTD) 5α-cholestan-3β-ol (purity 98%), bis (trimethylsilyl) trifluoroacetamide (BSTFA) with 1% of trimethylchlorosilane (TMCS), heptane, dichloromethane (DCM) and methanol (all GC grade) were sourced from Sigma-Aldrich Co. (Arklow, Co. Wicklow, Ireland). Pyridine was supplied by Santa Cruz Biotechnology, Inc. (Dallas, TX, USA). Deionised water (DI) was sourced from a Millipore Elix 15 water purification system (Merck Millipore, Darmstadt, Germany).

### 2.2. Trimethylsilyl Ether Derivatives Preparation

To quantify cholesterol, a hot saponification with subsequent derivatisation to trimethylsilyl ether derivatives similar to that described in Grasso, Brunton, Monahan and Harrison [8] was performed. Stock standard solutions for cholesterol and ISTD were prepared daily in DCM. In order to validate the method, an aliquot of the ISTD (for neat solutions 50 µL of 0.01 g/mL ISTD solution and for meat matrix effect 200 µL of 0.05 g/mL ISTD solution) and the stock standard solutions (final target concentrations for GC injection of 1, 5, 10, 15 and 20 µg/mL DCM) were added to 50 mL polypropylene centrifuge tubes (Sarstedt, Nurmbrecht, Germany) and dried with nitrogen. To test recoveries and matrix effects only, 0.5 g of turkey meat was added to each test tube. For both neat solutions and tubes containing meat sample, 30 mL of 4 M methanolic potassium hydroxide were added and tube contents were homogenised with an Ultra Turrax T25 (Janke and Kunkel IKA-Labortechnik) at 9500 rpm for 30 s. Saponification (water bath at 60 °C for 1 h) and cholesterol extraction (3 × 5 mL heptane layers) was carried out similarly to Grasso et al. (2015). After the heptane extracts were dried under nitrogen, one in 20 dilution steps was carried out for meat containing samples. Briefly, the dried samples were transferred to 10 mL flasks and brought to volume using DCM, then a 0.5 mL aliquot was transferred to clean 10 mL Pyrex tubes and dried. For neat solutions (without meat), derivatisation was performed in the same tube immediately after drying. Derivatization was carried out, as reported by Laakso [10]. The derivatised samples in Pyrex tubes were then moved to 10 mL volumetric flasks and brought to volume with DCM to reach the final target concentrations for cholesterol (1, 5, 10, 15 and 20 µg/mL). An aliquot (1.5 mL) was then transferred to GC vials. Measurements were carried out in triplicate for the studies of linearity, precision and accuracy, and in duplicate to test for meat matrix effect and recoveries.

### 2.3. Gas Chromatographic Analysis

Trimethylsilyl ether derivatives were separated and quantified as described in [8] with a PerkinElmer Clarus 580 Gas Chromatograph (PerkinElmer, Waltham, MA, USA) fitted with a flame ionisation detector. The oven temperature programme used was described in Du and Ahn [11].

### 2.4. Method Validation

Linearity, precision and accuracy were validated in neat solutions (without meat), while matrix effects and recoveries were tested in the presence of a turkey meat product. Validation in meat was considered, but any meat matrix would have contained cholesterol. Removing cholesterol from the meat matrix would likely also remove other lipophilic compounds, altering the matrix significantly, therefore the validation in neat solution was preferred. A five-point calibration curve using neat solutions was performed in triplicate on three non-consecutive days (concentration range 1–20 µg cholesterol/mL DCM). Using these calibration curves, accuracy (how close the mean test results obtained by the method are to the true concentration of the analyte) and precision (how close the individual measures of an analyte are when the procedure is applied many times to several aliquots of a matrix) [9] were calculated and linearity was assessed. The acceptable limits for precision and accuracy were set as 20% relative standard deviation (RSD) at the LLOQ and 15% RSD for all other standards in accordance with those set by the FDA [9]. These solutions were also used to calculate the RRFs according to equation below, where PA is the peak area, and the concentrations are in μg/mL (Equation (1)):

Cholesterol RRF = (PA Cholesterol/Cholesterol concentration)/(PA ISTD/ISTD concentration).(1)

In addition, the recovery and matrix effects were calculated similar to An et al. [12], by spiking the meat samples at two levels (low = 3 µg/mL and high = 18 µg/mL) at two different time points: Once before saponification and once before derivatisation. The recovery was calculated by comparing the peak area for cholesterol when spiked before saponification (A) with the cholesterol peak area obtained when spiked before derivatisation (B). The matrix effect was calculated by comparing the absolute peak area for cholesterol in neat solution (C) with the peak area of cholesterol spiked before derivatisation (B) at the same concentrations. The calculations, from An, Lee and Jung [12], were as follows (Equations (2) and (3)):

Recovery (%) = (A/B) × 100,(2)

Matrix effect (%) = (B/C) × 100.(3)

Two quality control (QC) samples were prepared at the low and high end of the scale (in concentrations between points 1 and 2 (3 µg/mL) and between points 4 and 5 (18 µg/mL)) to verify accuracy and precision independently.

Calculations of linearity, accuracy, precision and recovery were made with Microsoft Excel 2010 (Microsoft, Redmond, WA, USA).

## 3. Results

### 3.1. Peak Separation and Instrument Performance

**Figure 1:**
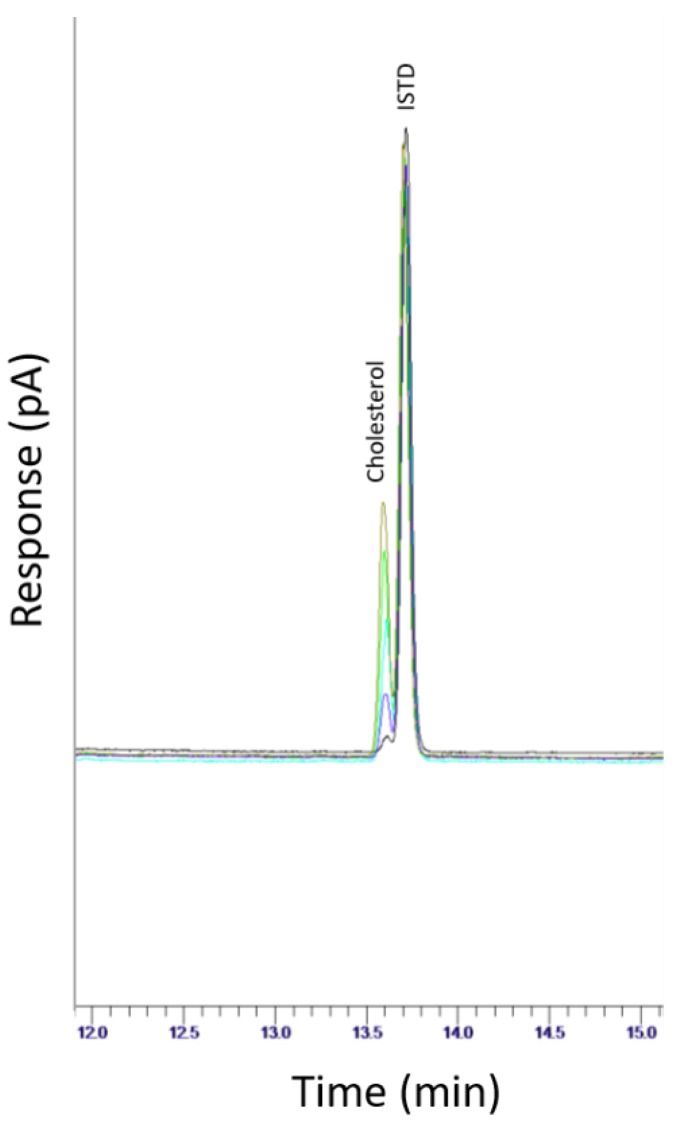
Overlay of all cholesterol standards in the calibration range considered (1–20 µg/mL).

A portion of a chromatogram showing an overlay of all standards in the calibration range considered (1–20 µg/mL) is shown in Figure 1.

Both ISTD and cholesterol eluted within 15 min and the compounds were sufficiently separated with the chromatographic conditions used. Instrument performance was suitable, achieving a% RSD ≤1.9% for each run.

### 3.2. Linearity

Varying amounts (1, 5, 10, 15 and 20 µg/mL) of cholesterol standard in the presence of a constant amount of ISTD were analysed in triplicate using the same procedure described in Section 2.2 on three different days. The intercepts and correlation coefficients of the three individual calibration curves were for replicate 1 intercept 0.0212 with *r*^2^ of 0.998, for replicate 2 intercept 0.0203 with *r*^2^ of 0.998, for replicate 3 intercept 0.0189 with *r*^2^ of 0.999. A plot of the meat of all three reps had intercept 0.0201 with *r*^2^ of 0.991. The individual calibration curves show good linearity for cholesterol (*r*^2^ ≥ 0.998).

### 3.3. Precision, Accuracy, and RRF

The precision, calculated as % RSD of the cholesterol peak area ratios on each day, met the FDA validation criteria, with precision ≤4.8% RSD on each of the three days of analysis (Table 1).

Accuracy fulfilled the FDA validation criteria for all the concentration levels tested, within ± 10% of nominal at the LLOQ and ± 7% at the other concentrations (replicate 1 is given as an example in Table 2).

The RRFs of cholesterol varied slightly across the three days (rep 1 = 1.053, rep 2 = 1.012, rep 3 = 0.944) and the mean RRF across the three days was 1.001. Considering the RRF, Equation (4) is used to calculate the cholesterol content in the sample in mg/g. In this equation PA is the peak area, the weights are in grams, the ISTD purity is in decimal notation (e.g., 0.99 for 99% purity), the RRF is 1.001 and 20 is the dilution factor.

Cholesterol (mg/g) = (PA cholesterol/PA ISTD) × (weight ISTD/weight sample) × (ISTD purity/RRF) × 20.(4)

Table 2 shows the accuracy for cholesterol determination measured in cholesterol standard solutions using the equation from the calibration curve (on the day), the overall equation (resulting from the three curves), the RRF (on the day) and the overall RRF for rep 1.

### 3.4. Recovery, Matrix Effect, and QC

**Figure 2:**
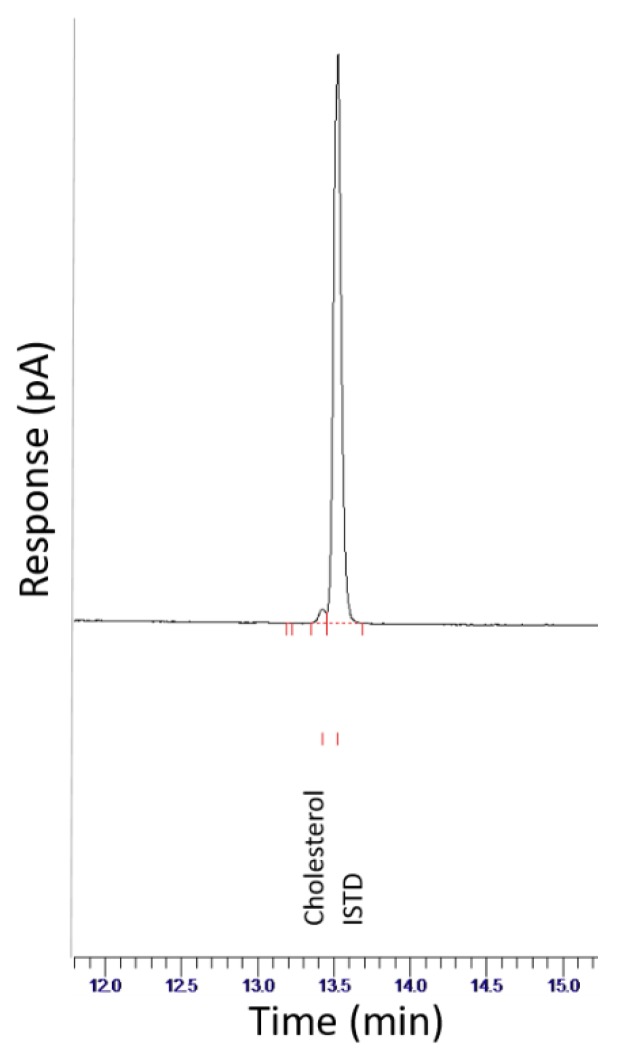
Overlay of all cholesterol standards using meat samples.

The results of the recovery and matrix effect calculations are presented in Table 3 and a sample chromatogram is shown in Figure 2.

Recovery across the three reps ranged between a minimum of 93.6% and a maximum of 115.1%. This is positive, as no recovery correction factors are needed to account for losses [13].

## 4. Discussion

This method was shown to be both accurate and precise when used to analyse cholesterol in a turkey meat product in the range of 0.4–8 mg cholesterol/g, without encountering any recovery or matrix effects. This range could also allow cholesterol determination in other raw meats and meat products with similar cholesterol content. Satisfactory peak separation, instrument performance and linearity were achieved. The use of RRFs, which are constant and not affected by day to day variations, makes it unnecessary to run a calibration curve with every run. Additionally, when 5α-cholestan-3β-ol is used as an ISTD for cholesterol quantification, adopting RRFs can improve accuracy. In fact, while in this case very little difference between the RF of cholesterol versus the IS was observed (i.e., RRF close to 1), this is not always the case. For example recently Grasso, Brunton, Monahan and Harrison [8] reported that the RRFs of three chemically similar plant sterols differed considerably to each other when 5α-cholestan-3β-ol was used as the ISTD (Campesterol = 1.0167, Stigmasterol = 1.4458, β-Sitosterol = 0.9029) while also being considerably different to that of cholesterol described here. This is important, because as mentioned in [8], other methods have either not used RRF when using 5α-cholestan-3β-ol as an ISTD [10], or they have used theoretical RFs (based on the chemical composition) when 5α-cholestan-3β-ol was used as ISTD [14].

No significant matrix effect was found with the addition of turkey meat. This clearly highlights the robustness of the method, as both a very low and a very high spike were recovered from the matrix, indicating that there is no matrix effect and that the approach of performing the validation in neat solution is justifiable.

## 5. Conclusions

An easy, quick and sensitive GC method for the determination of cholesterol in turkey meat products was validated. Calculation of RRF for the analysis of cholesterol will permit researchers to accurately determine cholesterol without the need for preparation daily calibration curves. This method could also be used together with our previously developed one [8] to allow for the parallel analysis of enriched levels of sterols in the same sample.

## Figures and Tables

**Table 1:** Precision for cholesterol measured on three different days at five concentrations using % RSD of cholesterol peak area ratios.

Concentration (µg/mL)	Precision (% Relative Standard Deviation)		
	Replicate 1	Replicate 2	Replicate 3
1	4.8	3.5	1.3
5	0.5	0.8	2.4
10	4.4	2.3	1.5
15	0.3	0.5	0.5
20	2.3	1.1	0.8
Quality Control 1	1.2	0.4	2.8
Quality Control 2	1.0	1.8	1.4

Quality Control 1 = 3 µg/mL and Quality Control 2 = 18 µg/mL.

**Table 2:** Accuracy for cholesterol measured using on the day and overall equations and RRFs (replicate 1 only).

Concentration (µg/mL)	Rep 1 Accuracy			
	Equation (on the Day)	Equation (Overall)	RRF (on the Day)	RRF (Overall)
1	104.3	110.0	104.0	109.43
5	96.7	102.0	96.5	101.48
10	100.4	105.8	100.1	105.32
15	99.2	105.6	98.9	104.06
20	100.7	106.2	100.5	105.69
Quality Control 1	96.3	101.6	96.1	101.1
Quality Control 2	101.5	107.0	101.2	106.5

Rep: Replicate; RRF: Relative response factor; Quality Control 1 = 3 µg/mL and Quality Control 2 = 18 µg/mL.

**Table 3:** Cholesterol recovery and matrix effect using meat samples spiked with high (18 µg/mL) and low (3 µg/mL) levels of cholesterol.

	Concentration	Rep 1	Rep 2	Rep 3	Average
Recovery	Low	115.1	101.3	99.5	105.3
	High	94.9	96.1	93.6	94.9
Matrix	Low	104.4	98.8	103.5	102.2
effect	High	112.6	92.9	89.7	98.4
